# Enhancing Drug Solubility, Bioavailability, and Targeted Therapeutic Applications through Magnetic Nanoparticles

**DOI:** 10.3390/molecules29204854

**Published:** 2024-10-13

**Authors:** Yue Zhuo, Yong-Gang Zhao, Yun Zhang

**Affiliations:** 1 School of Biomedical Science and Engineering, South China University of Technology, Guangzhou 511442, China; zhuoyue1102@gmail.com; 2 College of Biological and Environmental Engineering, Zhejiang Shuren University, Hangzhou 310015, China; 3 School of Materials Science and Engineering, NingboTech University, Ningbo 315100, China

**Keywords:** biological variability, nanotechnology, magnetic nanoparticles, drug absorption, drug solubility, targeted drug delivery, therapeutic efficacy, drug design

## Abstract

Biological variability poses significant challenges in the development of effective therapeutics, particularly when it comes to drug solubility and bioavailability. Poor solubility across varying physiological conditions often leads to reduced absorption and inconsistent therapeutic outcomes. This review examines how nanotechnology, especially through the use of nanomaterials and magnetic nanoparticles, offers innovative solutions to enhance drug solubility and bioavailability. This comprehensive review focuses on recent advancements and approaches in nanotechnology. We highlight both the successes and remaining challenges in this field, emphasizing the role of continued innovation. Future research should prioritize developing universal therapeutic solutions, conducting interdisciplinary research, and leveraging personalized nanomedicine to address biological variability.

## 1. Introduction

Biological variability represents one of the most formidable challenges in the realm of therapeutics and drug design, with profound implications for both patient outcomes and the efficacy of treatments [1]. This variability is not a mere statistical anomaly but a fundamental characteristic of human biology, arising from the complex interplay of genetic, physiological, and environmental factors [2]. These individual differences create a broad spectrum of drug responses, complicating the development of treatments that are consistently effective across diverse patient populations. To address this challenge, it is essential to delve deeply into the patient-specific factors that underlie variability in drug response, as the traditional one-size-fits-all approach in pharmacotherapy often fails to meet the needs of all patients [3].

At the core of this challenge is the genetic diversity among individuals, which can significantly influence drug metabolism and response [4]. Genetic variations, such as polymorphisms in genes encoding enzymes like cytochrome P450, can lead to substantial differences in how drugs are metabolized, resulting in variability in both efficacy and safety profiles [5]. For instance, some individuals may metabolize a drug too quickly, rendering it ineffective, while others may metabolize it too slowly, leading to toxic levels in the body. This genetic variability is further compounded by physiological factors, such as age, sex, and organ function, all of which can influence drug absorption, distribution, metabolism, and excretion. Moreover, environmental factors—including diet, lifestyle, and concurrent medications—add another layer of complexity, as they can alter pharmacokinetics and pharmacodynamics, making drug response even more unpredictable [1].

In this intricate landscape, understanding the interplay between physiological parameters and the intrinsic properties of drugs becomes crucial [3]. One key aspect of this interplay is drug absorption, which is significantly influenced by the gastrointestinal (GI) environment. Variations in GI pH, for example, can have a profound impact on drug solubility and, consequently, on bioavailability. These variations highlight the importance of developing innovative drug formulations that can overcome the challenges posed by biological diversity. Traditional drug formulations, which assume a relatively uniform patient population, may fail to provide optimal therapeutic outcomes in the face of such variability [1].

Given the pivotal role of biological variability in drug response, it is imperative to explore how this variability intersects with drug solubility—a critical determinant of bioavailability and therapeutic efficacy [5]. Drug solubility is not only influenced by genetic and environmental factors but also by physiological conditions that vary among individuals. These factors can lead to significant differences in drug dissolution and absorption rates, which in turn affect therapeutic outcomes and patient safety [2]. Poor solubility is a common barrier in drug development, as it can severely limit a drug’s absorption, leading to unpredictable therapeutic levels and increasing the risk of adverse reactions [4]. For example, drugs with low solubility often exhibit poor GI absorption, which can result in erratic plasma concentrations that may exceed the therapeutic threshold, triggering harmful side effects.

Research has consistently shown that addressing solubility limitations is critical for improving drug efficacy and safety. Studies have demonstrated that enhancing drug solubility through advanced formulation techniques can lead to more consistent absorption rates, thereby stabilizing plasma drug levels within the therapeutic window [6]. This stabilization is essential for reducing the risk of side effects and ensuring that the drug exerts its intended therapeutic effect [5]. The importance of solubility in drug development cannot be overstated, as it directly impacts the balance between therapeutic benefits and potential risks [2].

The challenge of improving drug solubility and managing biological variability has spurred the exploration of innovative solutions, with nanotechnology emerging as a particularly promising approach. Nanotechnology, with its ability to manipulate materials at the molecular and atomic levels, offers new possibilities for enhancing drug solubility and delivery. Among the various nanotechnological approaches, magnetic nanoparticles (MNPs) have garnered significant attention for their potential to revolutionize drug delivery systems. These nanoparticles can be engineered to penetrate biological membranes more effectively and deliver drugs in a controlled manner, thereby ensuring higher bioavailability and more consistent therapeutic effects across diverse patient populations [5].

Nanotechnology, particularly the use of MNPs, represents a cutting-edge frontier in pharmaceutical sciences [6]. These nanoparticles offer unique advantages due to their controllable size, surface characteristics, and magnetic properties, which allow for precise targeting and controlled drug release within the body. By engineering nanoparticles to respond to external magnetic fields, it is possible to direct them to specific sites within the body, such as areas of inflammation or tumor sites. For example, in research by Naz et al., it was discovered that inorganic nano-drug delivery platforms, including MNPs, improve therapeutic performance at tumor sites by enabling precise targeting and effective drug delivery, minimizing off-target effects [7]. Similarly, Panahi et al. demonstrated that functionalized magnetic nanoparticles offer controlled drug release in inflammation treatment, providing an efficient means of targeting affected areas while minimizing impact on surrounding healthy tissues [8]. This targeted approach not only enhances the efficacy of drug delivery but also minimizes the systemic side effects associated with traditional therapies [5]. Furthermore, the surface of MNPs can be modified with various functional groups to improve their interaction with drug molecules, enhancing drug loading and solubility. These modifications also enable the nanoparticles to navigate the complex environment of the GI tract, overcoming the variability in pH levels that can affect drug solubility and absorption [3].

The strategic application of nanotechnology and MNPs holds great promise not only for enhancing drug efficacy but also for mitigating the effects of biological variability [2]. By providing a more consistent and targeted delivery mechanism, these technologies offer the potential to tailor treatments to individual patient needs, advancing a personalized approach to medicine. This personalized approach could significantly improve therapeutic outcomes, offering new hope for patients who have previously struggled with the limitations of conventional drug therapies [6].

In conclusion, the intersection of biological variability and drug solubility presents significant challenges but also opportunities for innovation in drug design and therapeutics. As research continues to uncover the complexities of these interactions, it becomes increasingly clear that the future of medicine lies in the ability to tailor treatments to the unique characteristics of each patient. Nanotechnology, particularly through the use of MNPs, represents a powerful tool in this endeavor, offering the potential to enhance drug delivery and efficacy in ways that were previously unimaginable. As we continue to explore and develop these technologies, we move closer to a future where personalized medicine becomes the standard of care, ensuring that each patient receives the most effective treatment possible [1]. In this review, we have conducted a comprehensive search of scientific databases, including PubMed, Scopus, and Web of Science. Using keywords such as “drug solubility”, “bioavailability”, “biological variability”, “nanotechnology”, and “magnetic nanoparticles”, we retrieved 101 articles published between 1995 and 2024. These articles were carefully selected based on their relevance to drug solubility challenges and innovative nanotechnological solutions for improving bioavailability. Our review focuses on the most recent advancements in addressing solubility limitations and variability in drug response.

## 2. Drug Solubility and Biological Variability

Drug solubility is a fundamental aspect of pharmacokinetics that directly influences the absorption, distribution, metabolism, and excretion (ADME) of a drug, ultimately determining its therapeutic efficacy. The solubility of a drug dictates its ability to dissolve in bodily fluids, a prerequisite for crossing biological membranes and entering systemic circulation. Therefore, understanding and optimizing drug solubility is crucial for developing effective therapeutic agents, particularly in the face of biological variability that can further complicate drug absorption and response.

### 2.1. Impact of Drug Solubility on Absorption and Therapeutic Outcomes

The solubility of a drug is a primary determinant of its bioavailability—the proportion of an administered dose that reaches the systemic circulation in an active form. Poor solubility is often the root cause of poor bioavailability, which can result in subtherapeutic concentrations of the drug at the target site, thereby diminishing its efficacy. For oral drugs, solubility plays an especially critical role, as the drug must dissolve in the GI fluids before it can be absorbed into the bloodstream [1]. Consequently, drugs with low solubility may require higher doses to achieve therapeutic levels, increasing the risk of side effects and toxicity.

Several factors influence drug solubility, including the drug’s chemical properties, such as molecular weight, polarity, and crystalline structure. These properties determine how well a drug can interact with and dissolve in a solvent, such as water or lipid-based media in the GI tract [3]. Furthermore, advanced drug formulation techniques—such as the use of surfactants, prodrugs, and nano-formulations—can modify solubility and improve therapeutic outcomes. For example, Quercetin, a bioactive compound with poor water solubility, has been reformulated using nano-delivery systems to enhance its solubility and bioavailability, addressing the limitations of its conventional formulations [9].

The impact of solubility on therapeutic outcomes cannot be underestimated. Drugs with low solubility may exhibit erratic absorption, leading to unpredictable therapeutic levels in the body. This unpredictability complicates dosing regimens and can result in therapeutic failure or adverse effects. For instance, Felodipine, Ketoprofen, and Ibuprofen are biopharmaceutics classification system (BCS) Class II drugs that exhibit poor bioavailability due to their low solubility. Recent studies have shown that incorporating these drugs into metal-organic frameworks (MOFs) significantly enhances their solubility and, consequently, their therapeutic efficacy [10]. Similarly, Apixaban, an anticoagulant, suffers from low solubility, which limits its bioavailability. Research has demonstrated that combining Apixaban with Quercetin in a cocrystal form can significantly improve the solubility and absorption of both drugs [11]. On the other hand, enhancing drug solubility can improve absorption, leading to more predictable pharmacokinetic profiles and more consistent therapeutic effects [1]. Nano- and microemulsions, for example, have been used to improve the solubility and bioavailability of drugs for treating depression and anxiety, addressing the variability in absorption and brain bioavailability [12]. By improving solubility, it is possible to stabilize plasma drug levels within the therapeutic window, reducing the risk of side effects and enhancing efficacy.

Thus, the development of formulations that improve solubility is not merely a technical challenge but a vital component of successful drug design.

### 2.2. Linking Physiological pH Conditions to Drug Solubility and GI Absorption

The GI tract presents a complex and variable environment where pH levels fluctuate significantly across different regions, affecting drug solubility and, consequently, drug absorption. The pH of the GI tract ranges from highly acidic in the stomach (pH 1–3) to more neutral or slightly alkaline in the small intestine (pH 5–7) [13]. This variation in pH can have profound effects on the solubility of drugs, particularly those with pH-dependent solubility.

Drugs that are weak bases are generally more soluble in the acidic environment of the stomach, where they exist predominantly in their ionized form. Conversely, weak acids are more soluble in the more basic environment of the small intestine, where they also exist in their ionized form. However, the solubility of many drugs can decrease sharply as they move from one region of the GI tract to another, leading to variable absorption and bioavailability [14]. For instance, a drug that is highly soluble in the stomach may precipitate out of solution as it enters the small intestine, reducing the amount available for absorption.

The pH solubility profile of a drug is, therefore, crucial in predicting its absorption and therapeutic efficacy. Drugs with pH-dependent solubility may have variable absorption depending on the site of release within the GI tract. This variability can lead to fluctuations in plasma drug levels, which in turn can affect the consistency of therapeutic outcomes. To address these challenges, formulation strategies such as enteric coating, which protects the drug from the acidic environment of the stomach, can be employed [15]. This allows the drug to dissolve and be absorbed in the more favorable pH conditions of the intestine.

Moreover, the use of buffering agents or pH modifiers in drug formulations can help maintain the drug in its soluble form across different regions of the GI tract, enhancing its absorption and bioavailability. These strategies are particularly important for drugs with narrow therapeutic windows, where precise control over absorption is necessary to maintain therapeutic efficacy while minimizing the risk of toxicity [16].

### 2.3. Exploring Case Studies: Variability in Drug Efficacy Due to Solubility Challenges

Case studies provide valuable insights into the practical challenges of drug solubility and its impact on therapeutic outcomes. The following examples illustrate how solubility issues can lead to variability in drug efficacy and how innovative formulation strategies can address these challenges.

Dasatinib and Sorafenib are examples of kinase inhibitors where solubility challenges have directly impacted their bioavailability and therapeutic outcomes. Both drugs suffer from low solubility, leading to pharmacokinetic variability across patients. To address this issue, researchers have developed novel formulations using amorphous solid dispersion technology. This advancement significantly improved the bioavailability of these drugs while reducing variability, thus optimizing the therapeutic outcomes for a broader range of patients [17].

Itraconazole, an antifungal agent with notoriously poor water solubility, has also been the subject of innovative drug delivery research. A self-nano-emulsifying drug delivery system (SNEDDS) has been developed to enhance its solubility and bioavailability. This formulation demonstrated significant improvements in drug stability and content, offering a solution to the variability in drug absorption associated with conventional formulations [18].

Ivacaftor, a drug used for the treatment of cystic fibrosis, previously exhibited variability in absorption due to its sensitivity to food intake and its low solubility. By developing an SNEDDS formulation using a novel oil phase, researchers have been able to reduce food effects and inter-individual variability in absorption, significantly enhancing the bioavailability of Ivacaftor [19].

These case studies underscore the critical role of solubility in drug development and the need for innovative formulation strategies to address variability in drug absorption and efficacy. By improving solubility, it is possible to enhance bioavailability, reduce variability in therapeutic outcomes, and ultimately improve patient safety and treatment success [20].

## 3. Nanotechnology in Drug Design

### 3.1. Harnessing Nanotechnology: Enhancing Drug Solubility and Delivery

Nanotechnology represents a revolutionary approach to drug design, offering novel solutions to long-standing challenges such as poor drug solubility and inadequate bioavailability. By manipulating materials at the nanoscale—typically between 1 and 100 nanometers—nanotechnology enables the development of drug delivery systems that can overcome biological barriers and improve therapeutic outcomes.

One of the primary advantages of nanotechnology in drug design is its ability to enhance the solubility of poorly water-soluble drugs. Traditional drug formulations often struggle with achieving adequate solubility, leading to low bioavailability and reduced therapeutic efficacy. Nanotechnology addresses this issue by reducing the particle size of the drug, thereby increasing the surface area available for dissolution. This, in turn, enhances the drug’s solubility in biological fluids, facilitating better absorption and more consistent therapeutic effects [21].

**Figure 1:**
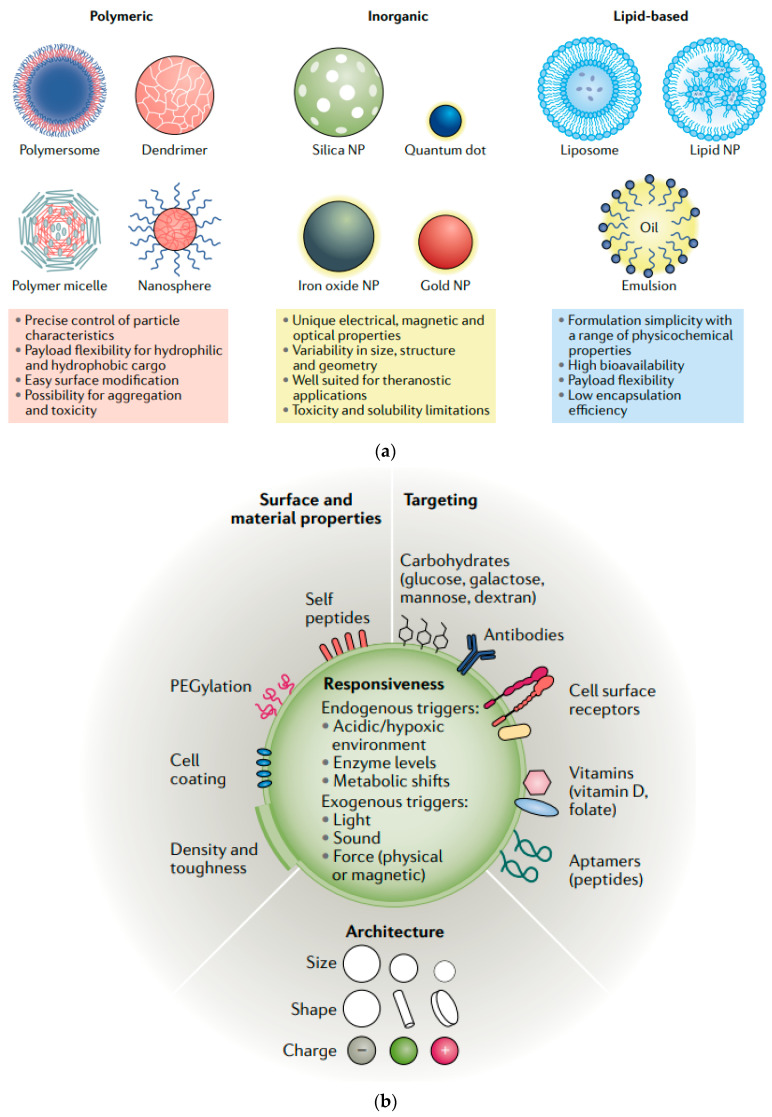
Classes of NPs: each class of NPs has numerous broad advantages and disadvantages regarding cargo, delivery, and patient response (**a**) and commonly engineered NP surface properties that allow for enhanced solubility and delivery (**b**). Reprinted with permission from Ref. [5]. Copyright (2021) Springer Nature.

Nanocarriers, such as liposomes, polymeric nanoparticles, dendrimers, and solid lipid nanoparticles, are commonly used to improve the solubility and delivery of drugs [22]. These carriers can encapsulate hydrophobic drugs, protecting them from degradation in the GI tract and enhancing their solubility in the aqueous environment of the body. For instance, liposomes—spherical vesicles composed of lipid bilayers—can encapsulate both hydrophilic and hydrophobic drugs, improving their solubility and stability. Similarly, polymeric nanoparticles can be engineered to release drugs in a controlled manner, improving the solubility and delivery of drugs and ensuring sustained therapeutic levels over time (Figure 1) [5].

Beyond solubility enhancement, nanotechnology also offers significant advantages in drug delivery. Nanocarriers can be engineered to target specific tissues or cells, reducing systemic side effects and improving the therapeutic index of drugs. Targeted delivery is particularly important in treating diseases such as cancer, where conventional therapies often damage healthy tissues. Nanoparticles can be functionalized with ligands that recognize and bind to specific receptors on the surface of target cells, ensuring that the drug is delivered precisely where it is needed [23]. This targeted approach not only improves the efficacy of the drug but also minimizes the risk of adverse effects.

Moreover, nanotechnology enables the design of multifunctional drug delivery systems that can combine diagnostic and therapeutic functions—so-called “theranostics”. These systems can be used to monitor drug delivery in real-time, allowing for adjustments in treatment regimens based on the patient’s response [24]. This level of precision in drug delivery is expected to play a crucial role in the development of personalized medicine, where treatments are tailored to the individual characteristics of each patient.

**Figure 2:**
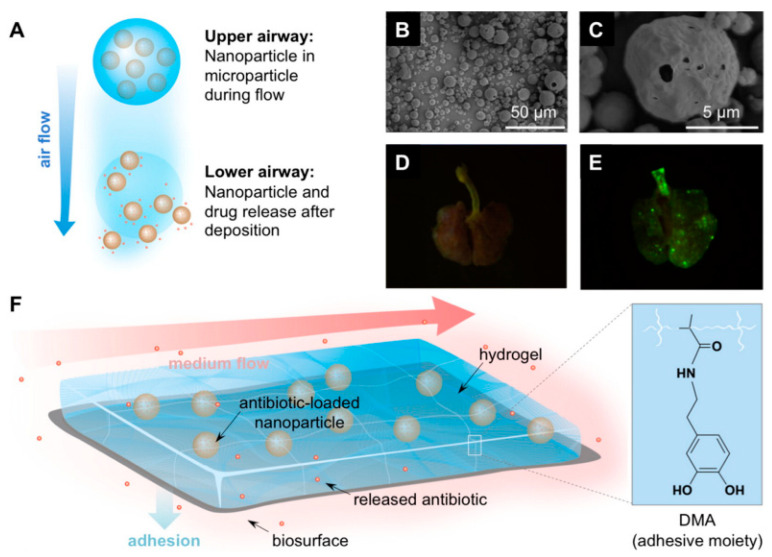
(**A**) Schematic illustration of inhalable microparticles as carriers for delivery of drug-loaded nanoparticles to the deep lung. (**B**) Scanning microscopic images of microparticles made from mannitol and leucine using a spray-drying process. The microparticles were loaded with nanoparticles made from glyceryl monostearate and soybean phosphatidylcholine with a double emulsion process. (**C**) A zoomed-in image of (**B**). (**D**) Fluorescence images of lungs from untreated rat and (**E**) rat after intrapulmonary delivery of microparticles fluorescein-labeled nanoparticles. (**F**) Schematic illustration of a nanoparticle–hydrogel hybrid (NP–gel) system with tissue adhesive properties for localized antibiotic delivery under flow conditions. In this design, dopamine methacrylamide (DMA) containing catechol functional group was conjugated into gel matrix for adhesion. Reprinted with permission from Ref. [25]. Copyright (2017) Elsevier B.V.

The potential of nanotechnology in enhancing drug solubility and delivery is supported by a growing body of research. Recent advancements have demonstrated the effectiveness of nanocarriers in improving the bioavailability of poorly soluble drugs and in delivering them to specific sites within the body. For example, research by Gao et al. (2019) highlighted the potential of nanotechnology in the oral delivery of poorly soluble anticancer drugs, where nanoparticles improved both the solubility and bioavailability of the drugs, leading to better therapeutic outcomes (Figure 2) [25].

In a word, nanotechnology offers a transformative approach to overcoming the challenges of poor drug solubility and delivery. By enabling the development of nanoscale drug delivery systems, it is possible to enhance the solubility, stability, and targeting of drugs, thereby improving their therapeutic efficacy and safety. As research in this field continues to advance, nanotechnology is poised to play a pivotal role in the future of drug design and personalized medicine.

### 3.2. Engineering MNPs for Improved Solubility and Targeted Delivery in the GI Tract

MNPs represent a cutting-edge approach in the field of drug delivery, particularly for addressing challenges related to drug solubility and targeted delivery within the GI tract. Their unique properties, including magnetic responsiveness, controllable size, and surface modifiability, make them highly suitable for enhancing drug solubility and ensuring precise drug delivery to specific sites within the body. In this section, we delve into how MNPs can be engineered to improve drug solubility and facilitate targeted delivery within the GI tract, drawing upon recent research and developments in the field.

MNPs are typically composed of magnetic cores, such as iron oxide, coated with biocompatible materials like polymers, lipids, or inorganic substances. These coatings not only stabilize the nanoparticles but also offer opportunities for further functionalization, such as the attachment of targeting ligands, drug molecules, or imaging agents [26]. The magnetic properties of these nanoparticles allow them to be manipulated using external magnetic fields, enabling controlled navigation and accumulation at specific sites within the body.

#### 3.2.1. Enhancing Drug Solubility Using MNPs

Drug solubility is a critical factor that directly influences the bioavailability and therapeutic efficacy of pharmaceutical compounds. The challenge of poor solubility is particularly pronounced for drugs designed to act in the GI tract, where varying pH levels and complex biological environments can hinder effective drug absorption. In recent years, the use of MNPs has emerged as a promising strategy to overcome these solubility challenges, offering a means to enhance drug solubility, stability, and, ultimately, therapeutic efficacy.

**Figure 3:**
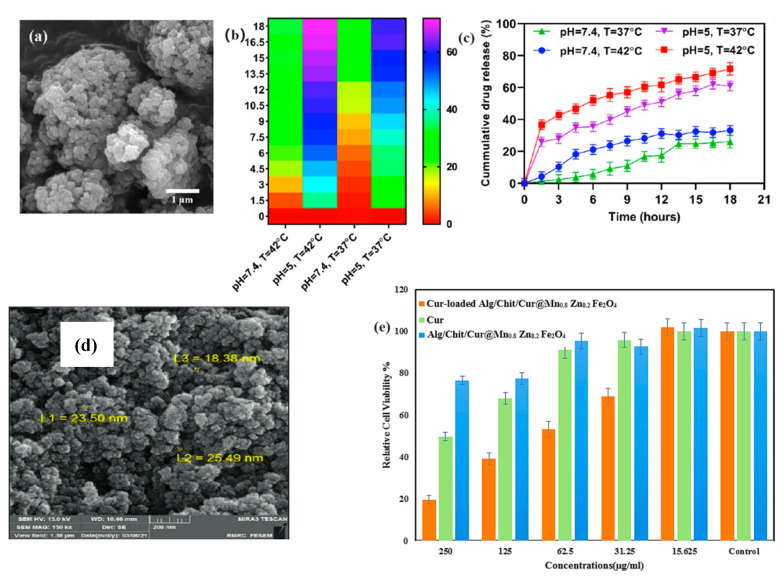
SEM image of Fe_3_O_4_@SiO_2_@CS-g-PNVCL (**a**), release profiles of the hydrocortisone drug at different pH levels (5, 7.4) and temperatures (37, 42 °C) (**b**,**c**). Reprinted with permission from Ref. [27]. Copyright (2023) American Chemical Society; FE-SEM image of Alg/Chit/Cur@Mn_0.8_Zn_0.2_Fe_2_O_4_ (**d**), Cell viability of breast cancer cells with different amounts of Cur-loaded Alg/Chit@Mn_0.8_Zn_0.2_Fe_2_O_4_, Cur, and Alg/Chit@Mn_0.8_Zn_0.2_Fe_2_O_4_ for 48 h (**e**). Reprinted with permission from Ref. [28]. Copyright (2023) Springer Nature.

MNPs have unique properties that make them ideal candidates for improving drug solubility. Their small size, large surface area, and the ability to be functionalized with various coatings enable them to interact more effectively with poorly soluble drugs, facilitating better dispersion and solubility in aqueous environments. For instance, a study demonstrated the successful use of MNPs, specifically PLA-HA/Fe_3_O_4_, loaded with curcumin, to improve the drug’s solubility in the GI tract. This formulation showed potential in treating colorectal cancer by enhancing the bioavailability of curcumin, a compound known for its low solubility and bioavailability [26]. Moreover, another study explored the use of CS-g-PNVCL-coated Fe_3_O_4_@SiO_2_ core–shell nanoparticles as an efficient drug delivery system for poorly soluble drugs. The results indicated significant improvement in drug solubility and therapeutic efficacy, highlighting the potential of such MNP-based systems to enhance bioavailability and reduce the dosage required for effective treatment (Figure 3a) [27].

The mechanisms by which MNPs enhance drug solubility are multifaceted. These nanoparticles can act as carriers that disperse the drug in the GI tract, preventing aggregation and improving the wettability of hydrophobic drug molecules. Functionalization of MNPs with biocompatible polymers like chitosan and sodium alginate further enhances these effects by providing additional stability and controlled release profiles. For example, a study on chitosan and sodium alginate-functionalized MNPs loaded with curcumin showed a significant increase in water solubility and drug release, leading to enhanced therapeutic efficacy against breast cancer cells (Figure 3b) [28].

**Figure 4:**
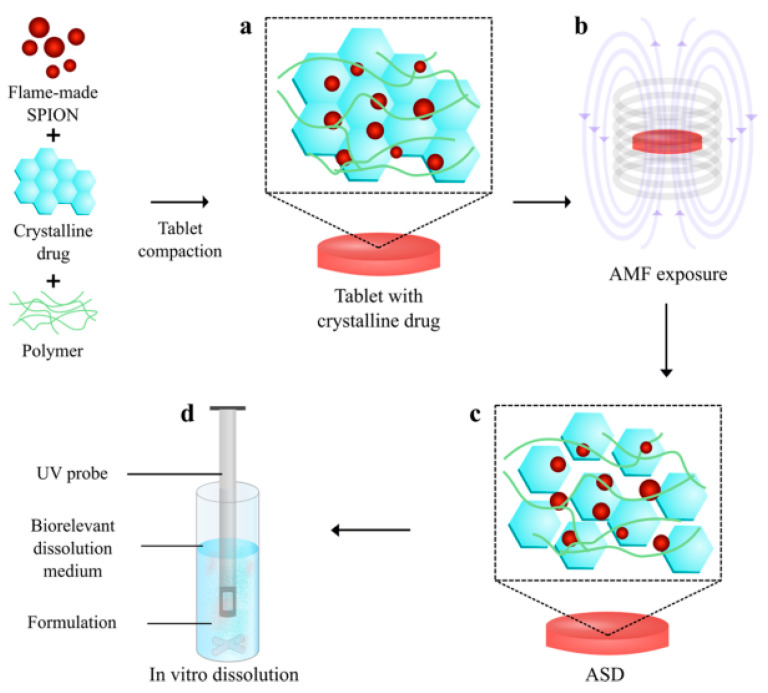
Amorphization by magnetic hyperthermia of a poorly aqueous soluble, crystalline drug in a tablet. (**a**) Crystalline drug is compacted into tablets with flame-made doped SPIONs and a polymer. (**b**) Tablets are exposed to an AMF to form an (**c**) ASD. (**d**) Performance of the ASD is evaluated by an in vitro dissolution assay. Reprinted with permission from Ref. [29]. Copyright (2022) American Chemical Society.

Additionally, MNPs can facilitate the amorphization of drugs, a process where the crystalline structure of a poorly soluble drug is converted into an amorphous form, which has higher solubility. This was demonstrated in a study where hyperthermia-induced in situ amorphization by superpara MNPs significantly increased the solubility of a model drug in the GI tract, showcasing the potential of MNPs in solubility enhancement (Figure 4) [29]. The practical applications of MNPs in enhancing drug solubility are evident in various studies focused on specific drug formulations. For instance, magnetic ferrite nanoparticles coated with bovine serum albumin and glycine polymers have been developed for the controlled release of curcumin. This approach not only improved the solubility of curcumin in the GI tract but also provided a stable and sustained release profile, which is crucial for maintaining therapeutic levels over time [30].

Another compelling example is the development of magnetic nanostructured lipid carriers (MNLC) co-loaded with Docetaxel, a chemotherapy drug, and MNPs. This formulation significantly enhanced the solubility of Docetaxel, reducing its toxicity and improving its therapeutic efficacy in lung cancer treatment [31].

#### 3.2.2. Targeted Delivery within the GI Tract

The GI tract presents a complex and challenging environment for drug delivery, especially for therapeutic agents that require precise targeting and controlled release. MNPs have emerged as a promising tool for achieving targeted drug delivery within the GI tract, offering significant advantages in terms of enhancing drug absorption, reducing systemic side effects, and improving therapeutic outcomes. This section explores the application of MNPs in targeted delivery, focusing on their ability to navigate the GI environment, their functionalization for specific targeting, and their use in clinical applications.

**Figure 5:**
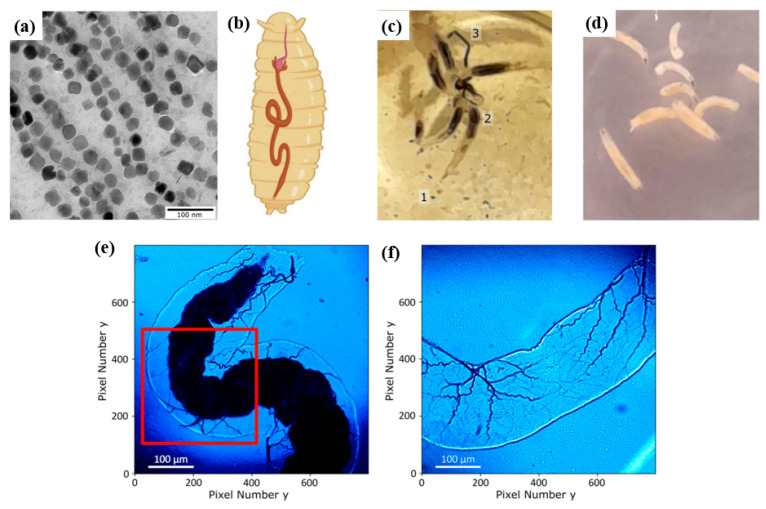
Transmission electron microscopy image of the magnetic nanoparticles (**a**), sketch of the GI tract in a Drosophila larva (**b**), picture of Drosophila larvae exposed for 24 h to MNPs: the MNPs show a dark color and can be observed (1) in suspension in the PBS as dots scattered in the well, (2) inside the GI tract of the larva and (3) excreted as feces (**c**), picture of Drosophila larvae exposed to the 24 h PBS control: the average length of Drosophila melanogaster 3rd instar larvae is approximately 4 mm (**d**), transmission light image of the measured sample (the intestine organ is clearly visible, and the dark area is generated by the accumulated MNPs blocking the light transmission, the area corresponding to the magnetic measurements is indicated by a red square) (**e**), transmission light image of a control intestine organ without MNPs (**f**). Reprinted with permission from Ref. [32]. Copyright (2024) Royal Society of Chemistry.

MNPs are uniquely suited for targeted drug delivery within the GI tract due to their responsiveness to external magnetic fields. This property allows for the precise control of drug-loaded MNPs, enabling them to be guided to specific locations within the GI tract, where they can release their therapeutic payload. For example, nitrogen-vacancy center magnetic imaging was used to track Fe_3_O_4_ nanoparticles within the GI tract of Drosophila melanogaster larvae, demonstrating the feasibility of using MNPs for targeted delivery in the GI tract (Figure 5) [32].

The integration of MNPs with advanced technologies further enhances their targeting capabilities. A study on liquid–metal soft electronics coupled with multi-legged robots illustrated how MNPs could be used in conjunction with robotic systems to achieve targeted drug delivery in the GI tract. This approach significantly improved the ability to overcome obstacles within the GI tract and allowed for long-term tracking of the nanoparticles, highlighting the potential of MNPs in complex GI environments [33].

**Figure 6:**
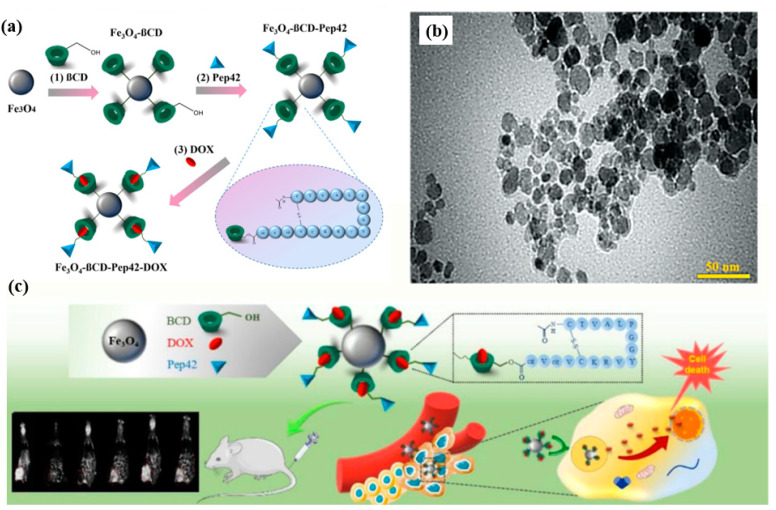
Consecutive steps for the formation of Fe_3_O_4_-ßCD-Pep42-DOX (**a**), transmission electron microscopy image of Fe_3_O_4_-ßCD-Pep42 NPs (**b**), and the potential capability of Fe_3_O_4_-ßCD-Pep42-DOX as a multifunctional nanoplatform in cancer therapy (**c**). Reprinted with permission from Ref. [35]. Copyright (2023) American Chemical Society.

One of the key strategies for achieving targeted delivery within the GI tract is the functionalization of MNPs with ligands that can recognize and bind to specific receptors on the surface of target cells. This approach not only enhances the specificity of drug delivery but also improves the therapeutic efficacy of the drug. For instance, glucosamine-modified mesoporous silica-coated MNPs were developed for the targeted delivery of methotrexate to cancer cells in the GI tract. This functionalization allowed the nanoparticles to effectively target tumor cells, demonstrating significant therapeutic potential in both in vitro and in vivo models [34]. Similarly, Pep42-targeted iron oxide MNPs functionalized with β-cyclodextrin and loaded with doxorubicin were shown to have potent anticancer activity against GI cancer cells. The targeted delivery system improved the uptake of the drug by cancer cells while minimizing its impact on healthy cells, thus reducing the side effects commonly associated with doxorubicin therapy (Figure 6) [35].

The application of MNPs in targeted drug delivery within the GI tract has been explored in various clinical contexts, with promising results. For example, silibinin-loaded magnetic niosomal nanoparticles (MNNPs) were developed for the treatment of colorectal cancer. This targeted delivery system enhanced the cytotoxicity of silibinin against colorectal cancer cells, showing increased cellular uptake and minimal toxicity to normal cells. These findings suggest that MNNPs could be an effective and safe option for targeted therapy in colorectal cancer [36]. Moreover, folic-acid-conjugated magnetic triblock copolymer nanoparticles were designed for the dual-targeted delivery of 5-fluorouracil to colon cancer cells. This system demonstrated enhanced cellular uptake by cancer cells, improved antitumor efficacy, and reduced toxicity compared to non-targeted formulations. The success of such targeted systems underscores the potential of MNPs in improving the outcomes of chemotherapy for GI cancers [37].

Another innovative application involved the development of a novel magnetically actuated robotic capsule for site-specific drug delivery within the GI tract. This capsule was designed to navigate the complex GI environment and deliver drugs directly to specific tissues, thereby enhancing the efficacy of the treatment and minimizing systemic exposure. Such advancements in MNP-based drug delivery systems highlight the ongoing innovation in this field and its potential to revolutionize the treatment of GI diseases [38].

#### 3.2.3. Engineering Considerations for MNPs in Drug Delivery

MNPs have garnered significant attention in the field of drug delivery due to their unique properties, including their responsiveness to external magnetic fields, high surface area, and the ability to be functionalized for targeted delivery. However, the successful application of MNPs in drug delivery systems requires careful engineering to address challenges related to biocompatibility, drug loading efficiency, controlled release, and the physical interactions between MNPs and biological environments. This section discusses critical engineering considerations for the design and optimization of MNPs in drug delivery applications.

**Figure 7:**
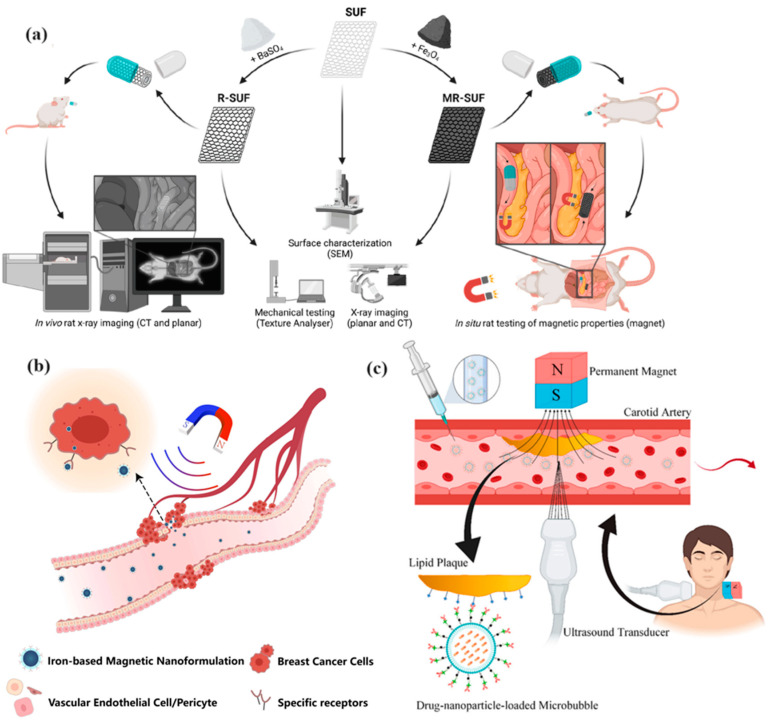
Self-unfolding foils (SUFs) (M/R-) functionalized by incorporating BaSO_4_ or Fe_3_O_4_ nanoparticles for improving their applicability within oral drug delivery (**a**). Reprinted with permission from Ref. [40], Copyright (2023) American Chemical Society. Schematic diagram of targeted drug delivery for magnetothermal therapy (**b**). Reprinted with permission from ref. [39], Copyright (2024) Dove Medical Press Ltd. Deviation of the drug carriers toward the target site as a result of external forces (**c**). Reprinted with permission from Ref. [45], Copyright (2021) Taylor & Francis.

One of the primary engineering challenges in the development of MNPs for drug delivery is the need to optimize their surface properties to enhance biocompatibility, reduce toxicity, and improve their interaction with target cells. Surface engineering often involves coating MNPs with biocompatible materials, such as polymers or proteins, which can provide a stable interface between the nanoparticles and biological tissues. For example, a recent study highlighted the importance of surface engineering in magnetic iron oxide nanoparticles used for breast cancer diagnostics and drug delivery. By modifying the surface properties of these nanoparticles, researchers were able to enhance their physicochemical characteristics, making them more suitable for both diagnostic imaging and therapeutic applications (Figure 7b) [39].

Functionalization of MNPs with targeting ligands, such as antibodies or peptides, is another critical aspect of surface engineering. This approach allows MNPs to selectively bind to specific cell receptors, enhancing the precision of drug delivery and minimizing off-target effects. For instance, a study on the functionalization of self-unfolding foils with MNPs demonstrated improved retention and controlled motion within the GI tract, suggesting that functionalization can significantly enhance the efficacy of oral drug delivery systems (Figure 7a) [40].

The design of MNP-based drug delivery systems also requires careful consideration of the physical and chemical interactions between the nanoparticles and the biological environment. Mathematical modeling plays a crucial role in predicting these interactions and optimizing the design of MNPs. For example, a study on magnetic scaffolds (MagSs) used as drug delivery systems identified several challenges, such as inhomogeneous drug distribution and burst release, which were attributed to a lack of proper modeling and optimization. The researchers proposed a new modeling framework using the Gompertz equation and the Korsmeyer–Peppas model to address these issues, improving the design and utilization of MagSs for drug delivery applications [41].

Another critical aspect of optimization involves the consideration of hemodynamic variables in drug delivery systems that utilize MNPs. For instance, hybrid nanoparticles have been used to optimize blood flow efficiency in stenosed arteries, where magnetic fields can be employed to reduce pressure within the stenosis and enhance drug delivery. This approach underscores the importance of integrating physical principles, such as fluid dynamics and magnetism, into the design of MNP-based drug delivery systems [42].

Effective drug delivery systems must achieve high drug loading efficiency and controlled release to ensure therapeutic efficacy and patient safety. The engineering of MNPs for drug delivery often involves the development of dual-responsive systems that can respond to multiple stimuli, such as pH and temperature, to trigger drug release at the desired site and time. A study on the optimization of MNPs for engineering erythrocytes as theranostic agents highlighted the importance of encapsulating superparamagnetic iron oxide nanoparticles (SPIONs) within red blood cells to overcome rapid clearance from circulation, thereby enhancing drug delivery efficiency and therapeutic outcomes [43].

Moreover, the development of multifunctional MNPs that combine magnetic properties with other therapeutic modalities, such as hyperthermia, offers a promising approach to enhancing drug delivery systems. For example, a study on the fabrication of biocompatible magnetic nickel ferrite-supported fluorapatite nanoparticles demonstrated their potential for combined drug delivery and hyperthermia treatment, highlighting the importance of integrating multiple functionalities into a single nanoparticle system to achieve better therapeutic outcomes [44].

Finally, engineering considerations must account for the interactions between MNPs and the biological environment to ensure safety and efficacy. This includes understanding how MNPs interact with cells, tissues, and biological fluids, as well as assessing their long-term biocompatibility and potential toxicity. For instance, studies have shown that the magnetic properties of MNPs can be optimized to enhance their adhesion to specific tissues or cells, such as atherosclerotic plaques, under the influence of magnetic and ultrasound fields. This highlights the importance of optimizing both the magnetic properties of the nanoparticles and the external stimuli used to guide them in the body (Figure 7c) [45].

For all MNPs mentioned in this section, here is a table showing the name of the drug or compound with poor solubility whose solubility and bioavailability have been improved, type of magnetic nanoparticles, MNP size, route of administration, effect or remark on increasing overall bioavailability or therapeutic outcomes (Table 1).

### 3.3. Breakthroughs in Drug Formulations: Success Cases with MNPs

MNPs have emerged as a powerful tool in the realm of drug delivery, particularly for enhancing the solubility and targeting of drugs that are otherwise challenging to administer [46]. The past few years have witnessed significant advancements in the development of MNP-based drug delivery systems, leading to several successful formulations that are either in clinical trials or already being used in practice. This section provides an in-depth review of current research progress and presents examples of successful drug formulations that utilize MNPs, illustrating their potential to revolutionize therapeutic strategies [47].

#### 3.3.1. Advances in MNPs Synthesis and Functionalization

MNPs have become a cornerstone in the development of advanced drug delivery systems, particularly due to their unique magnetic properties that allow for controlled movement and targeted delivery under the influence of external magnetic fields. Recent advances in the synthesis and functionalization of MNPs have significantly enhanced their efficacy, safety, and versatility in drug delivery applications. This section explores some of the notable breakthroughs in MNP synthesis and functionalization, highlighting how these advancements are paving the way for more effective and targeted therapeutic interventions.

The synthesis of MNPs has evolved considerably, with new techniques enabling better control over particle size, morphology, and surface properties. One of the most widely used methods for synthesizing MNPs is the co-precipitation method, which has been further refined to produce highly uniform and stable nanoparticles. For example, a study demonstrated the synthesis of magnetite nanoparticles using the co-precipitation method, followed by a silica coating. This approach not only preserved the magnetic properties of the nanoparticles but also enhanced their suitability for drug delivery by providing a biocompatible surface that facilitates movement in a liquid medium [48].

Microfluidic technologies have also emerged as a powerful tool for the synthesis of lipid-based nanoparticles, including MNPs. These technologies offer precise control over the synthesis process, allowing for the production of nanoparticles with highly uniform size distributions and tailored surface properties. This precision is crucial for ensuring the consistent behavior of MNPs in drug delivery applications, particularly in achieving targeted delivery and controlled release [49].

Surface functionalization is a critical aspect of MNP design, as it determines the nanoparticles’ interaction with biological environments and their ability to target specific cells or tissues. Recent advancements in surface functionalization have focused on enhancing the biocompatibility, stability, and targeting capabilities of MNPs. For instance, the functionalization of MNPs with biocompatible polymers, such as PEGylation, has been shown to improve their circulation time in the bloodstream and reduce immunogenicity. This functionalization is particularly important in applications where prolonged circulation is needed to achieve effective targeting of tumors or other pathological sites [50].

Functionalization with targeting ligands, such as antibodies or peptides, has also been a major focus of recent research. These ligands enable MNPs to selectively bind to specific receptors on target cells, thereby improving the precision of drug delivery. For example, curcumin-loaded mesoporous silica nanoparticles functionalized with folic acid demonstrated enhanced anticancer activity and biocompatibility, showcasing the potential of such functionalized MNPs in targeted cancer therapy [51].

**Figure 8:**
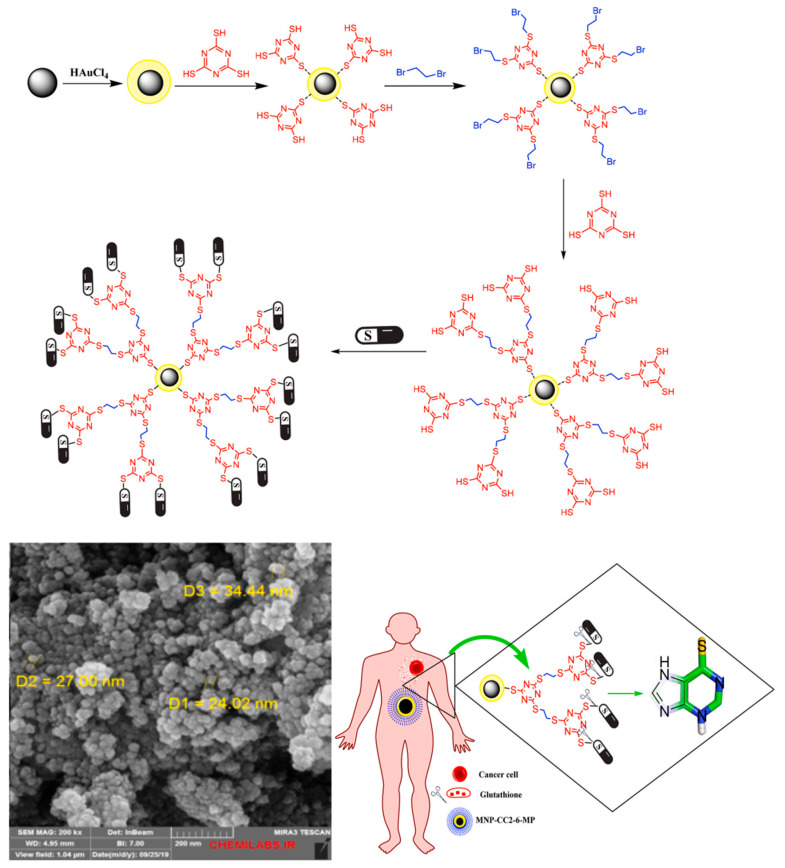
Gold-coated MNPs decorated with 6-mercaptopurine for targeted drug delivery. Reprinted with permission from Ref. [52], Copyright (2023) Elsevier B.V.

Another exciting development in the field of MNPs is the creation of multifunctional nanoparticles that combine several therapeutic modalities into a single platform. These nanoparticles can be engineered to deliver drugs, provide diagnostic imaging, and even facilitate hyperthermia treatment. For example, a study on gold-coated MNPs decorated with a thiol-containing dendrimer demonstrated their potential for targeted drug delivery, hyperthermia treatment, and enhancement of MRI contrast agents. This multifunctionality not only improves the therapeutic efficacy of the nanoparticles but also reduces the need for multiple treatment interventions (Figure 8) [52].

The integration of magnetic properties with other functionalities, such as pH or temperature sensitivity, has also been explored to achieve more responsive drug delivery systems. These stimuli-responsive nanoparticles can release their therapeutic payload in response to specific environmental triggers, such as the acidic environment of a tumor or the application of an external magnetic field. Such advancements are crucial for developing next-generation drug delivery systems that can offer more precise and controlled therapeutic interventions [53].

#### 3.3.2. MNPs in Cancer Therapy

MNPs have emerged as a powerful tool in cancer therapy due to their unique properties, including their ability to be guided by external magnetic fields, their high surface area-to-volume ratio, and their potential for functionalization with therapeutic agents. These properties make MNPs ideal candidates for targeted drug delivery, magnetic hyperthermia, and imaging-guided therapy. This section explores the latest advances in the use of MNPs for cancer therapy, highlighting their mechanisms of action, clinical applications, and ongoing challenges.

One of the key advantages of MNPs in cancer therapy is their ability to be directed to specific tumor sites using external magnetic fields. Once localized, these nanoparticles can be used in various therapeutic modalities, including drug delivery, hyperthermia, and ferroptosis—a form of iron-dependent cell death. Recent advances have focused on designing MNPs that can supply iron ions to promote ferroptosis, providing a novel approach to cancer treatment. These MNPs can also be used in conjunction with diagnostic magnetic resonance imaging (MRI), enabling image-guided therapy that combines diagnosis and treatment in a single platform [54].

Magnetic hyperthermia is another promising application of MNPs in cancer therapy. In this approach, MNPs are directed to the tumor site and then subjected to an alternating magnetic field (AMF), which causes the nanoparticles to generate localized heat. This heat can induce cell death in cancerous tissues while sparing surrounding healthy tissue. For instance, a study demonstrated that magnetic iron oxide nanoparticles clad with tannic acid were effective in inhibiting cancer cell growth through localized hyperthermia when activated by visible laser light [55].

MNPs also play a crucial role in enhancing the precision and efficacy of drug delivery systems. By functionalizing MNPs with specific ligands, such as antibodies or peptides, researchers can target the nanoparticles to cancer cells with high specificity. This targeted approach minimizes the systemic side effects typically associated with conventional chemotherapy. For example, a recent study developed nylon-6-coated magnetic nanocomposites and nanocapsules loaded with doxorubicin, a widely used chemotherapeutic agent. These nanoparticles exhibited excellent loading capacity and pH-sensitive drug release properties, making them highly effective for targeted cancer treatment [56].

The development of dual-targeted nanoparticles has further enhanced the therapeutic potential of MNPs. For instance, dual-targeted ECO/siDANCR nanoparticles were developed for the treatment of triple-negative breast cancer, a particularly aggressive form of cancer. These nanoparticles were designed to deliver siRNA specifically to cancer cells, thereby inhibiting the expression of oncogenic long noncoding RNAs (lncRNAs) and suppressing tumor growth. The inclusion of MRI capabilities in these nanoparticles also allowed for real-time monitoring of the treatment process [57].

MNPs have shown promise in various clinical applications, particularly in the treatment of difficult-to-treat cancers. For example, a study on paclitaxel-loaded lipid-coated MNPs demonstrated their effectiveness in the dual chemo-magnetic hyperthermia therapy of melanoma. This combined therapy not only reduced the size of the tumors but also minimized the systemic side effects associated with conventional chemotherapies, highlighting the potential of MNPs to improve patient outcomes [58].

Another innovative application involves the use of superparamagnetic iron oxide nanoparticles (SPIONs) for the treatment of pancreatic cancer. When combined with proton therapy and hyperthermia, SPIONs significantly enhanced the cytotoxic effects on pancreatic tumor cells, leading to improved therapeutic efficacy. This synergistic approach underscores the potential of MNPs to be integrated into multimodal cancer therapies that offer a more comprehensive treatment strategy [59].

#### 3.3.3. MNPs in Neurological Disorders

MNPs have increasingly become a focal point in the treatment of neurological disorders due to their ability to traverse the blood–brain barrier (BBB), their potential for targeted drug delivery, and their use in various neuromodulation techniques. This section explores the recent advances in the application of MNPs in neurological disorders, discussing their therapeutic mechanisms, clinical applications, and the challenges that lie ahead.

MNPs hold significant promise in the treatment of neurological disorders through their ability to be guided by external magnetic fields, allowing for precise targeting within the central nervous system (CNS). One innovative approach involves the use of magnetic microhydrogels, which have shown superior neuron activation capabilities compared to traditional superparamagnetic iron oxide nanoparticles (SPIONs). These microhydrogels can be precisely controlled, enabling more effective neuromodulation and potentially offering new treatment options for neuropsychiatric disorders [60].

**Figure 9:**
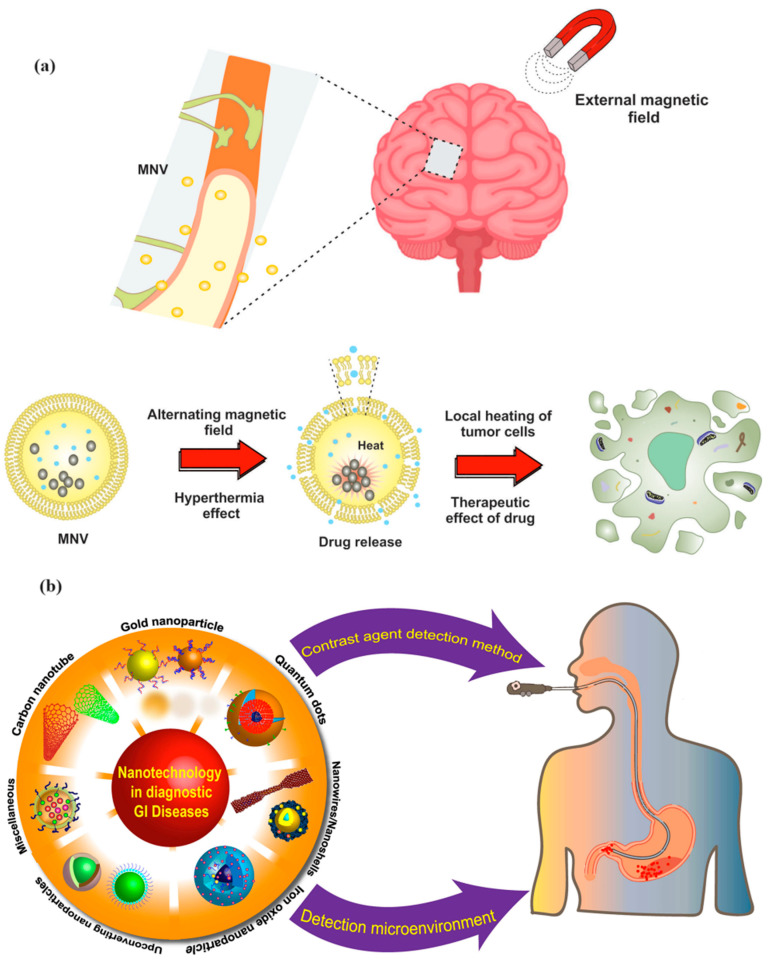
Magnetically targeted NVs for CNS imaging and drug delivery, and hyperthermia-triggered release of loaded drug (**a**). Reprinted with permission from Ref. [61], Copyright (2024) Taylor & Francis. Application of nanoparticles in the diagnosis of GI diseases (**b**). Reprinted with permission from Ref. [64], Copyright (2023) Taylor & Francis.

Another notable development is the use of magnetic nanovesicles (MNVs), which combine the benefits of magnetic targeting with the controlled release of therapeutic agents. These MNVs are designed to improve the diagnosis and treatment of CNS disorders by increasing the efficiency of drug delivery to specific brain regions while minimizing side effects. This approach is particularly beneficial in managing conditions such as Alzheimer’s disease and Parkinson’s disease, where targeted delivery is crucial for effective treatment (Figure 9a) [61].

The clinical potential of MNPs extends beyond basic research, with several studies demonstrating their efficacy in treating complex neurological conditions. For instance, magnetothermal nanoparticle technology has been shown to alleviate Parkinsonian-like symptoms in mice by providing therapeutic effects similar to deep brain stimulation without the need for invasive surgery. This technology utilizes MNPs that generate localized heat when subjected to an alternating magnetic field, thereby modulating neural activity and reducing symptoms associated with Parkinson’s disease [62].

In addition to Parkinson’s disease, MNPs have also been explored for their potential in treating neurodegenerative diseases like Alzheimer’s disease. Superparamagnetic iron oxide nanoparticles (SPIONs) have been utilized to label extracellular vesicles derived from human forebrain organoids. These labeled vesicles can be tracked using magnetic resonance imaging (MRI), providing a novel method for monitoring disease progression and the effectiveness of therapeutic interventions in real time [63].

#### 3.3.4. MNPs in GI Disorders

MNPs are emerging as a promising tool in the diagnosis and treatment of GI disorders due to their unique properties, such as the ability to be guided by external magnetic fields, high surface area for drug loading, and ease of functionalization. These attributes make MNPs highly suitable for targeted drug delivery, enhanced imaging, and therapeutic applications in GI diseases. This section explores the recent advances in the application of MNPs for managing GI disorders, highlighting their mechanisms of action, clinical applications, and the challenges involved.

MNPs play a crucial role in enhancing the diagnosis of GI disorders by improving the sensitivity and specificity of imaging techniques. For instance, nanoparticles integrated with advanced imaging methods have significantly improved the detection capabilities for GI tumors and inflammatory bowel diseases (IBD). These nanoprobes allow for early diagnosis and precise staging of GI disorders, which are critical for effective treatment planning. The use of MNPs in magnetic resonance imaging (MRI) has been particularly beneficial, as these nanoparticles serve as contrast agents, enhancing the visualization of inflammation and neoplastic lesions in the GI tract (Figure 9b) [64].

The role of MRI in diagnosing and monitoring bowel diseases has been further highlighted in recent studies, where it has proven invaluable in staging diseases like IBD and rectal cancer. MNPs, when used as MRI contrast agents, can provide detailed images that help in assessing disease activity, thereby aiding in the formulation of targeted treatment strategies [65].

Beyond diagnostics, MNPs are being developed for therapeutic purposes in GI disorders, particularly in the targeted delivery of drugs to specific sites within the GI tract. The encapsulation of therapeutic agents in MNPs allows for localized treatment, reducing systemic side effects and improving drug efficacy. For example, Fe_3_O_4_ nanoparticles encapsulated with orange pectin have shown significant potential in treating GI cancers. These nanoparticles exhibit strong anti-cancer properties against various GI cancer cell lines, demonstrating their potential as an effective treatment option [66].

Additionally, MNPs are being explored for their ability to address inflammation in the GI tract. The use of these nanoparticles in delivering anti-inflammatory drugs directly to the site of inflammation in conditions such as IBD could revolutionize the management of these chronic diseases. In a study by Giannousi et al. (2019), manganese ferrite nanoparticles functionalized with nonsteroidal anti-inflammatory drugs (NSAIDs) demonstrated significant anti-inflammatory effects in gastrointestinal inflammation, including IBD. The nanoparticles were shown to deliver the anti-inflammatory agents directly to the inflamed tissue, minimizing side effects and enhancing therapeutic outcomes [67].

MNPs hold significant potential in the diagnosis and treatment of GI disorders. Their ability to enhance imaging techniques, deliver targeted therapies, and improve patient outcomes makes them a promising tool in modern medicine. However, the challenges of safety, scalability, and regulatory approval must be addressed to fully realize the benefits of MNPs in clinical practice. Continued research and innovation in this field will be crucial in overcoming these hurdles and expanding the application of MNPs in GI healthcare.

#### 3.3.5. MNPs in Cardiovascular Disorders

MNPs have been utilized in the diagnosis and treatment of cardiovascular disorders due to their unique properties, including their ability to be guided by external magnetic fields, their high surface area for drug loading, and their ease of functionalization. This section explores the recent advances in the application of MNPs for managing cardiovascular disorders, highlighting their mechanisms of action, clinical applications, and the challenges involved.

One of the primary advantages of MNPs in cardiovascular disorders is their ability to enhance imaging techniques. Magnetic resonance imaging (MRI) has been significantly improved through the use of iron oxide nanoparticles (IONPs) as contrast agents, enabling detailed visualization of cardiovascular structures and functions. These nanoparticles enhance the contrast in MRI, making it easier to detect and monitor the progression of cardiovascular diseases. Furthermore, MNPs can be functionalized with targeting ligands, allowing for more precise imaging of atherosclerotic plaques and other cardiovascular abnormalities [68].

In addition to imaging, MNPs have shown potential in modulating vascular functions. For example, applying a magnetic field to endothelial cells can influence calcium ion channel activity and signaling pathways, which are crucial in maintaining vascular health and regulating blood pressure. This capability opens new avenues for treating vascular dysfunctions associated with aging and neurodegenerative disorders, where the regulation of neurovascular coupling is vital [69].

Beyond diagnostics, MNPs are being explored for their therapeutic potential in cardiovascular diseases, particularly in targeted drug delivery systems. The ability of MNPs to deliver therapeutics directly to the site of atherosclerotic plaques or other cardiovascular lesions reduces the systemic side effects commonly associated with cardiovascular drugs. This targeted approach enhances the efficacy of the treatment by concentrating the therapeutic agent at the disease site.

Recent advances in nanotheranostics have combined the diagnostic and therapeutic capabilities of MNPs into a single platform, offering personalized treatments for cardiovascular disorders. For instance, MNPs have been used to deliver drugs, genetic material, and imaging agents within nanocarriers, enabling both the treatment and monitoring of cardiovascular diseases in real time. This dual functionality not only improves patient outcomes but also allows for the continuous adjustment of therapy based on real-time feedback from diagnostic imaging [70].

Moreover, MNPs have been incorporated into flexible implantable devices designed for targeted treatment of cardiovascular diseases. These devices can aggregate MNPs at specific sites within the cardiovascular system, such as around atherosclerotic plaques, under the influence of an external magnetic field. This technology offers a non-invasive method for treating cardiovascular conditions, reducing the need for more invasive surgical interventions [71].

MNPs represent a transformative approach to diagnosing and treating cardiovascular disorders. Their ability to enhance imaging techniques, deliver targeted therapies, and improve patient outcomes makes them a promising tool in modern cardiology. However, the challenges of safety, scalability, and regulatory approval must be addressed to fully realize the benefits of MNPs in clinical practice. Continued research and innovation in this field will be crucial in overcoming these hurdles and expanding the application of MNPs in cardiovascular healthcare.

For all the applications of MNPs in the treatment of various diseases mentioned in this section, below is a summary table showing disease type, type of magnetic nanoparticles used, in vivo or in vitro models, and therapeutic outcomes (Table 2).

## 4. Challenges and Future Directions

The integration of nanotechnology and MNPs into drug design presents significant opportunities for enhancing drug delivery, improving therapeutic outcomes, and reducing biological variability in drug response. However, this promising field also faces several critical challenges that must be addressed to fully realize its potential in clinical applications. This section explores these challenges, the impact of nanotechnology on biological variability, and the future research directions necessary for advancing this technology.

### 4.1. Overcoming Hurdles in Integrating Nanotechnology and MNPs into Drug Design

The integration of nanotechnology, particularly MNPs, into drug design presents a promising frontier in the development of targeted therapies and advanced drug delivery systems. However, despite the potential, several significant challenges must be overcome to fully realize the benefits of MNPs in clinical applications. These challenges range from the complexities of nanoparticle synthesis and functionalization to the issues of biocompatibility, safety, and regulatory approval. This section delves into the key hurdles in integrating MNPs into drug design and explores potential solutions and future directions.

#### 4.1.1. Synthesis and Functionalization Challenges

One of the primary challenges in integrating MNPs into drug design is the complexity of their synthesis and functionalization. The precise control over the size, shape, and surface properties of MNPs is crucial for their effectiveness as drug carriers. The synthesis process must ensure uniformity in particle size and distribution, as these factors significantly influence the pharmacokinetics and biodistribution of the nanoparticles. For instance, a recent study highlighted the development of vinorelbine-loaded multifunctional MNPs, which demonstrated significant potential in anticancer drug delivery. However, the study also underscored the challenges in achieving the desired drug loading and release profiles due to the intricate nature of nanoparticle synthesis [72].

Functionalization of MNPs, which involves modifying their surface to enhance biocompatibility and targeting capabilities, presents another layer of complexity. The surface properties of nanoparticles, including surface charge, geometry, porosity, and functional groups, play a critical role in determining their interaction with biological systems. In particular, surface functionalization must be carefully tailored to avoid rapid clearance by the immune system and to ensure that the nanoparticles can effectively target specific tissues or cells. The importance of these factors was highlighted in a review that discussed the hemocompatibility of MNPs and their potential applications in regenerative medicine, cancer therapy, and drug delivery [73].

#### 4.1.2. Biocompatibility and Safety Concerns

Biocompatibility is a critical concern in the development of MNP-based drug delivery systems. Nanoparticles introduced into the body must not elicit an adverse immune response or cause toxicity in healthy tissues. Ensuring the biocompatibility of MNPs requires rigorous testing and optimization, particularly in terms of their interaction with blood components and other biological fluids. Studies have shown that the surface characteristics of nanoparticles, such as their coating materials and functional groups, significantly influence their hemocompatibility and overall safety in clinical applications.

One promising approach to enhancing the biocompatibility of MNPs involves the use of biocompatible coatings, such as polyethylene glycol (PEG) or natural polymers, which can reduce the likelihood of immune recognition and extend the circulation time of the nanoparticles in the bloodstream. Additionally, the development of stimuli-responsive coatings, which release their therapeutic payload in response to specific physiological triggers, offers a potential solution to the challenge of achieving controlled and targeted drug delivery. A study discussing the use of polypropylene sulfide-coated MNPs for cancer therapeutics highlighted the benefits of such coatings in improving biocompatibility and enabling responsive drug release under oxidative stress conditions [74].

#### 4.1.3. Regulatory and Manufacturing Challenges

The regulatory landscape for nanomedicine is complex and still evolving. The unique properties of MNPs, while advantageous for drug delivery, also pose challenges for regulatory approval. Nanoparticles often exhibit different pharmacokinetics and toxicology profiles compared to traditional drugs, necessitating extensive preclinical and clinical testing to ensure their safety and efficacy. Regulatory agencies require comprehensive data on the manufacturing process, quality control, and long-term effects of nanoparticles, which can be a significant hurdle for developers.

Moreover, the scalability of nanoparticle production remains a significant challenge. While laboratory-scale synthesis of MNPs is well-established, scaling up the production to meet industrial demands while maintaining consistency in quality and functionality is challenging. The transition from small-scale synthesis to large-scale manufacturing requires the development of robust and reproducible processes that can consistently produce nanoparticles with the desired properties. This challenge is compounded by the need for stringent quality control measures to ensure that the nanoparticles meet the necessary safety and efficacy standards for clinical use [75].

#### 4.1.4. Overcoming Biological Barriers

Another significant challenge in integrating MNPs into drug design is overcoming biological barriers, such as the blood–brain barrier (BBB) and the immune system. The BBB, in particular, presents a formidable obstacle to the delivery of therapeutic agents to the central nervous system (CNS). MNPs offer a potential solution by enabling targeted drug delivery across the BBB, but this requires precise control over the size and surface properties of the nanoparticles to ensure they can penetrate the barrier without causing damage or being cleared by the immune system.

To address these challenges, researchers are exploring various strategies, such as the use of magnetic targeting to guide MNPs across the BBB and the development of nanoparticles that can mimic natural transport mechanisms. A study on the use of SPIONs (superparamagnetic iron oxide nanoparticles) as drug carriers for CNS and inner ear disorders demonstrated the potential of MNPs to overcome these barriers but also highlighted the need for further research to optimize their delivery and minimize potential side effects [76].

### 4.2. Reducing Biological Variability in Drug Response through Advanced Nanotechnologies

Biological variability in drug response is a significant challenge in drug design and therapeutic interventions, often leading to varied efficacy and safety outcomes across different patient populations. This variability can arise from numerous factors, including genetic differences, environmental influences, and individual health conditions. Advanced nanotechnologies offer promising solutions to reduce this variability, thereby enhancing the precision and effectiveness of therapeutic interventions. This section explores how these technologies are being leveraged to minimize biological variability in drug response and the potential implications for personalized medicine.

#### 4.2.1. Precision Drug Delivery Systems

One of the most effective ways to reduce biological variability in drug response is through precision drug delivery systems enabled by nanotechnology. MNPs, for instance, can be engineered to deliver therapeutic agents directly to target tissues or cells, reducing the variability associated with systemic drug distribution. This targeted approach not only enhances drug efficacy but also minimizes side effects by ensuring that the drug acts only where it is needed.

A recent study demonstrated the use of MNPs to improve the delivery of chemotherapeutic agents to tumor cells. By applying an external magnetic field, the researchers were able to guide the drug-loaded nanoparticles to the tumor site, significantly reducing the variability in drug response that is often seen in traditional chemotherapy. This precision targeting helps to ensure that all patients, regardless of genetic or physiological differences, receive the optimal therapeutic dose at the target site, thereby reducing the overall variability in treatment outcomes [77].

#### 4.2.2. Genetic Influence on Drug Response

Advanced nanotechnologies also play a critical role in addressing genetic factors that contribute to biological variability in drug response. For example, genetic polymorphisms in genes related to nitric oxide (NO) production have been shown to influence individual responses to certain drugs. By using nanotechnology to deliver NO donors or inhibitors in a controlled manner, it is possible to modulate the NO pathway more precisely, thereby reducing variability in drug response among individuals with different genetic backgrounds.

A study explored the genetic influence of NO pathway genes beyond eNOS (endothelial nitric oxide synthase), such as ARG1, ARG2, DDAH1, DDAH2, and VEGF, on drug response. By understanding these genetic factors, researchers can design nanoparticle-based drug delivery systems that account for individual genetic differences, leading to more personalized and effective pharmacotherapy [78].

#### 4.2.3. Computational Models and Nanotechnology Integration

The integration of advanced computational models with nanotechnology offers another promising avenue for reducing biological variability in drug response. Computational docking and machine learning models can predict how different drugs interact with their targets at the molecular level, allowing for the design of nanoparticles that enhance drug–target interactions. This approach not only improves the precision of drug delivery but also reduces the variability in therapeutic outcomes.

A study on the integration of molecular docking into anti-cancer drug response prediction models demonstrated that such computational tools could improve the accuracy of predicting drug responses across different cell lines. By incorporating these models into the design of nanoparticle-based drug delivery systems, it is possible to tailor therapies more precisely to individual patient profiles, thereby reducing variability in drug response [79]. Similarly, advances in pharmacokinetic modeling and computational approaches for nanoparticles have further enhanced the design and optimization of drug delivery systems, offering insights into their interactions with biological environments [80]. Additionally, mechanistic modeling has been employed to predict drug uptake in tumor cells, providing a framework for understanding drug resistance and enhancing the efficacy of nanoparticle-based drug delivery systems [81].

By incorporating computational modeling into the design process of nanomedicine, we can improve the precision and predictability of therapeutic outcomes, potentially revolutionizing personalized medicine [82].

#### 4.2.4. Personalized Nanomedicine

Personalized nanomedicine, which combines nanotechnology with personalized medicine, holds great potential for reducing biological variability in drug response. By analyzing individual patient data, including genetic, proteomic, and metabolomic profiles, it is possible to design nanoparticle-based therapies that are specifically tailored to the unique characteristics of each patient. This approach ensures that the drug is delivered at the right dose, to the right place, and at the right time, reducing the chances of adverse reactions and improving therapeutic efficacy.

For instance, a neural matrix factorization (NeuMF) model was developed to predict unknown responses of cell lines to drugs. This model achieved high prediction accuracy, demonstrating its potential to reduce biological variability in drug response by tailoring treatments to individual patient profiles. When integrated with nanotechnology, such models can guide the design of nanoparticles that deliver drugs more effectively to the intended targets, thereby reducing variability in drug response [83].

### 4.3. Charting the Future: Interdisciplinary Research and Innovation in Nanotechnology for Therapeutics

The integration of nanotechnology into therapeutics represents one of the most promising frontiers in modern medicine. This convergence of disciplines—ranging from materials science and molecular biology to data science and engineering—has opened new avenues for treating diseases with unprecedented precision and efficacy. As we chart the future of nanotechnology in therapeutics, interdisciplinary research and innovation will play a pivotal role in overcoming existing challenges and pushing the boundaries of what is possible in healthcare.

#### 4.3.1. The Role of Interdisciplinary Collaboration

Interdisciplinary collaboration is essential for advancing nanotechnology in therapeutics. The complexity of designing, synthesizing, and applying nanomaterials to clinical problems requires the integration of knowledge from various scientific fields. For instance, the development of bio-nanotechnology-based cancer therapeutics is an area where interdisciplinary efforts have been particularly fruitful. Recent advancements have shown that combining expertise in molecular biology, chemistry, and nanotechnology can lead to the creation of highly effective diagnostic and therapeutic strategies for cancer treatment. These innovations are paving the way for personalized medicine, where treatments are tailored to the genetic and molecular profiles of individual patients [84].

Moreover, interdisciplinary research is not just about combining different fields; it also involves integrating new technologies that can enhance the capabilities of nanotechnology in therapeutics. For example, the integration of artificial intelligence (AI) with nanotechnology is revolutionizing the field of nano-biosensors, particularly in detecting epigenetic changes. AI-driven data analysis allows for the rapid processing of complex datasets, enabling more accurate and timely detection of diseases. This synergy between AI and nanotechnology is transforming how we approach diagnostics and therapeutic monitoring, making treatments more precise and effective [85].

Another important example of interdisciplinary innovation is the use of “green” mechanochemical technology in drug delivery systems. This approach, pioneered by Prof. Weike Su at the Collaborative Innovation Center of Green Pharmaceuticals in China and Prof. Alexander Dushkin from the Institute of Solid State Chemistry and Mechanochemistry in Russia, emphasizes environmentally sustainable methods to improve drug solubility and efficacy. Their work on mechanochemically prepared nanocapsules of celery seed oil demonstrated that this technique significantly enhances water solubility and bioavailability while maintaining a green and efficient encapsulation process [86]. Green mechanochemical technology uses mechanical force rather than traditional chemical solvents, reducing environmental impact while producing highly efficient and stable drug delivery systems. This interdisciplinary approach, combining principles of chemistry, engineering, and pharmaceutical sciences, opens new avenues for the development of eco-friendly therapeutic solutions.

#### 4.3.2. Innovation in Nanotechnology for Cancer Therapeutics

Cancer therapeutics is one of the most active areas of research and innovation in nanotechnology. The ability to design nanoparticles that can selectively target cancer cells while sparing healthy tissues has revolutionized the treatment landscape. Recent studies have highlighted the potential of organic–inorganic nanotechnological platforms in delivering cancer therapeutics. These platforms leverage the unique properties of nanomaterials—such as their ability to penetrate biological barriers and their high surface area for drug loading—to enhance the efficacy of chemotherapy and reduce side effects.

The innovation in this area extends to the development of hybrid nanomaterials that combine organic and inorganic components, offering the best of both worlds in terms of biocompatibility and functionality. For example, the use of gold nanoparticles conjugated with drugs and targeting ligands has shown great promise in targeting and eradicating tumors in preclinical models. Such interdisciplinary innovations are crucial for translating these technologies from the lab to the clinic, where they can have a real impact on patient outcomes [87].

#### 4.3.3. Expanding the Scope of Nanotechnology in Therapeutics

While cancer therapeutics remains a primary focus, the scope of nanotechnology in medicine is rapidly expanding to other areas, including the treatment of non-oncologic diseases. Nanotechnology’s potential to enhance drug delivery and diagnostics in urogenital diseases, for instance, demonstrates its versatility. Nanomaterial-based products are being developed to improve the detection and treatment of conditions such as urinary tract infections and erectile dysfunction, where conventional therapies have limited efficacy. The interdisciplinary research driving these innovations is essential for addressing the unmet needs in these fields and improving patient care [88].

Furthermore, the application of nanotechnology in renewable energy is an area of growing interest that intersects with medical research in surprising ways. The use of nanomaterials to enhance energy production and storage could have implications for powering medical devices, particularly in resource-limited settings. Interdisciplinary research in this area could lead to the development of sustainable energy solutions that support the deployment of advanced medical technologies, making cutting-edge treatments more accessible worldwide [89].

#### 4.3.4. The Economic and Societal Impact of Nanotechnology

The economic and societal impact of nanotechnology is another critical aspect of its future development. As interdisciplinary research continues to drive innovation, the commercialization of nanotechnological products will play a key role in determining their success. The development of liposomal technologies for drug delivery, for example, has been driven by entrepreneurial teams that have secured funding and navigated the complex regulatory landscape to bring these innovations to market. The success of such endeavors underscores the importance of not only scientific innovation but also the business acumen and collaborative efforts needed to translate research into real-world applications [90].

In addition to their economic impact, nanotechnologies have the potential to address some of the most pressing health challenges of our time, including the need for more equitable healthcare access. By reducing the cost and complexity of treatments, nanotechnology could help bridge the gap between high-resource and low-resource settings, ensuring that more people benefit from advanced medical care.

#### 4.3.5. Future Directions in Interdisciplinary Research

Looking to the future, interdisciplinary research in nanotechnology for therapeutics will continue to evolve, driven by the need to address increasingly complex healthcare challenges. One promising direction is the further integration of AI and machine learning with nanotechnology to develop smarter, more responsive therapeutic systems. These systems could automatically adjust drug dosages based on real-time monitoring of patient responses, leading to more personalized and effective treatments.

Another critical area of future research is the development of nanotechnologies that can interact with the human body in more sophisticated ways, such as through the modulation of immune responses or the repair of damaged tissues. These advancements will require close collaboration between biologists, engineers, and clinicians to ensure that the technologies are safe, effective, and tailored to the needs of patients.

The future of nanotechnology in therapeutics is bright, with interdisciplinary research and innovation at its core. By bringing together diverse fields of knowledge and leveraging new technologies, researchers are opening up new possibilities for treating diseases with greater precision and efficacy. As we continue to explore the potential of nanotechnology, it is essential to maintain a collaborative approach that fosters innovation and ensures that these advancements benefit as many people as possible.

## 5. Conclusions

### 5.1. Nanotechnology and MNPs: Addressing Drug Solubility Amidst Biological Variability

Nanotechnology, particularly through the use of MNPs, offers a transformative approach to overcoming the challenges posed by drug solubility and biological variability. These factors significantly impact the efficacy of therapeutic agents, often leading to inconsistent outcomes across different patient populations. MNPs, with their unique properties, provide promising solutions to these issues by enhancing drug delivery, improving solubility, and ensuring more consistent pharmacokinetic profiles.

#### 5.1.1. Enhancing Drug Solubility with MNPs

Drug solubility is a critical factor in determining the bioavailability of therapeutic agents. Poor solubility can lead to insufficient drug concentrations at the target site, reducing efficacy and increasing the likelihood of side effects. Nanotechnology, particularly the use of MNPs, addresses this issue by improving the dispersion and stability of poorly soluble drugs. For example, MNPs have been employed to enhance the solubility of anticancer drugs, ensuring that these agents remain effective even at lower doses. A study demonstrated that the integration of MNPs into drug delivery systems could improve the pharmacokinetic and pharmacodynamic characteristics of drugs, ultimately maximizing their therapeutic potential [91].

Additionally, the versatility of MNPs allows for their functionalization with various coatings and ligands, further enhancing their ability to interact with and stabilize poorly soluble drugs. This capability is particularly important in the context of oral drug delivery, where physiological conditions in the GI tract can significantly affect drug solubility and absorption. For instance, mesoporous silica nanoparticles (MSNs) have been developed to improve the solubility of oral drugs, offering precise dose delivery to specific sites and enhancing overall drug absorption [92].

The continuous innovation in nanotechnology has led to the development of advanced MNPs that further enhance drug solubility and address biological variability. For instance, the use of crosslinked alginate nanoparticles has shown promise as a drug carrier due to their biocompatibility and controllable drug release properties. These nanoparticles can be engineered to release drugs in response to specific physiological conditions, such as pH changes, which are often associated with disease states. This targeted release not only improves drug solubility but also reduces the variability in drug response among different patients [93].

Moreover, systematic reviews of nanotechnology applications have highlighted the potential of MNPs in improving the solubility and reducing the toxicity of drugs like Amphotericin B. This antifungal drug is known for its poor solubility and significant side effects, but the integration of MNPs into its delivery system has been shown to enhance its solubility and reduce its adverse effects, making it more effective across diverse patient populations [94].

#### 5.1.2. Addressing Biological Variability

Biological variability, stemming from genetic differences, environmental factors, and individual health conditions, poses a significant challenge to the consistent efficacy of therapeutic agents. Nanotechnology, particularly through the use of MNPs, offers a strategy to mitigate this variability by enabling targeted drug delivery and controlled release mechanisms. By delivering drugs directly to the site of action, MNPs reduce the influence of systemic factors that contribute to variability in drug response.

For example, iron oxide MNPs have been utilized in various biomedical applications, including drug delivery, hyperthermia, and as MRI contrast agents. These applications demonstrate the potential of MNPs to address both solubility issues and biological variability by ensuring that drugs are delivered precisely where they are needed, minimizing off-target effects and improving overall therapeutic outcomes [95].

The integration of nanotechnology and MNPs into drug design offers a robust solution to the challenges of drug solubility and biological variability. By enhancing drug solubility and enabling targeted delivery, MNPs ensure more consistent therapeutic outcomes across diverse patient populations. Continued research and innovation in this field are essential to fully realize the potential of MNPs, paving the way for more effective and universally applicable therapeutic solutions.

### 5.2. The Imperative for Ongoing Innovation and Research to Develop Universal Therapeutic Solutions

Ongoing innovation and research in nanotechnology are essential for advancing therapeutic solutions that are effective across diverse patient populations. As diseases become more complex and the demand for personalized medicine grows, the need for continuous innovation in nanotechnology becomes increasingly critical. This section highlights the importance of sustained research efforts in nanotechnology to develop universal therapeutic solutions, focusing on the advances in various medical fields and the potential impact on global healthcare.

#### 5.2.1. Advancing Universal Therapeutic Solutions

One of the most compelling reasons for ongoing research in nanotechnology is its potential to develop universal therapeutic solutions that can be adapted to a wide range of diseases and conditions. For instance, recent advancements in nanotechnology have shown significant promise in cancer therapy, where nanoparticles are used to deliver drugs directly to tumor sites, minimizing side effects and improving treatment efficacy. Research into biomembrane-grafted dendrimer–polymeric conjugates, which target the p53 protein in cancer cells, exemplifies how innovative nanotechnology can lead to more effective and broadly applicable cancer treatments [96].

Moreover, the development of nanotechnology-based therapeutics for managing inflammatory diseases underscores the versatility of this field. By leveraging the unique properties of nanoparticles, researchers are creating anti-inflammatory strategies that improve the diagnosis and treatment of diseases characterized by inflammation. This approach not only enhances the effectiveness of existing therapies but also opens the door to new treatment modalities that can be tailored to individual patient needs while maintaining broad applicability [97].

#### 5.2.2. Addressing Complex Health Challenges

The complexity of diseases like cancer, neurodegenerative disorders, and cardiovascular diseases requires therapeutic solutions that are both innovative and adaptable. Nanotechnology offers the tools needed to address these challenges by enabling precise targeting, controlled drug release, and real-time monitoring of treatment efficacy. For example, the National Cancer Institute’s Alliance for Nanotechnology in Cancer has successfully integrated nanotechnology into oncology, leading to paradigm-shifting solutions for diagnosing and treating cancer. Such programs highlight the critical role of ongoing research in driving the development of universal therapeutic solutions that can be applied across different types of cancer and patient demographics [98].

In addition, the role of nanotechnology in developing treatments for infectious diseases, such as tuberculosis, demonstrates its potential to create universal solutions for global health. Nanoparticles are being explored for their ability to accurately identify mycobacterial strains and improve drug delivery in tuberculosis management. These advancements are crucial for combating multidrug-resistant strains of the disease and ensuring that effective treatments are available to all patients, regardless of geographic or socioeconomic barriers [99].

#### 5.2.3. Innovation Ecosystems and Collaborative Research

The emergence of innovation ecosystems in nanotechnology is another factor driving the development of universal therapeutic solutions. These ecosystems, characterized by the collaboration of diverse stakeholders, including academic institutions, industry, and government agencies, are crucial for fostering innovation and ensuring that research translates into practical applications. In Israel, for example, the development of nanotechnology has been supported by an ecosystem that encourages simultaneous competition and cooperation among researchers and companies. This collaborative environment has facilitated the emergence of groundbreaking nanotechnologies that have the potential to revolutionize healthcare on a global scale [100]. Similarly, “green” mechanochemical technology has emerged as a promising innovation in drug delivery systems, thanks to international collaboration. Pioneered by Prof. Weike Su and Prof. Alexander Dushkin, this approach is an example of how collaborative research can lead to eco-friendly and scalable solutions in nanotechnology [86].

#### 5.2.4. The Future of Nanotechnology in Therapeutics

As we look to the future, the continued evolution of nanotechnology will be essential for addressing the most pressing healthcare challenges. Institutions like the National Center for Nanoscience and Technology in China are at the forefront of this innovation, advancing cutting-edge technologies that integrate interdisciplinary research and development. These efforts are crucial for maintaining the momentum of innovation in nanotechnology and ensuring that new therapeutic solutions are not only effective but also accessible to a global population [101].

The imperative for ongoing innovation and research in nanotechnology cannot be overstated. As the field continues to evolve, the potential to develop universal therapeutic solutions that address a wide range of diseases and conditions will only grow. By fostering interdisciplinary collaboration, supporting innovation ecosystems, and maintaining a commitment to research, the medical community can ensure that the benefits of nanotechnology are realized in the form of effective, accessible, and universally applicable treatments.

In summary, addressing the challenges of drug solubility and bioavailability remains crucial for improving therapeutic outcomes, especially in the face of biological variability. The exploration of MNPs has proven to be a promising approach to overcoming these challenges by enhancing drug delivery and enabling targeted therapy. From improving solubility for poorly bioavailable drugs like Apixaban and Felodipine to enabling localized drug release in conditions such as inflammation and cancer, MNPs offer a versatile platform. Furthermore, interdisciplinary innovation, such as green mechanochemical technology, has emerged as a sustainable way to produce these nanoparticle systems, highlighting the growing need for eco-friendly solutions in drug development. Continued research into the functionalization of nanoparticles, their interaction with physiological systems, and advancements in nanotechnology will pave the way for personalized treatments that account for biological variability. Ultimately, by leveraging the unique properties of magnetic nanoparticles, future therapies can be better tailored to individual patient needs, reducing side effects and enhancing efficacy across a wide range of therapeutic areas.

## Figures and Tables

**Table 1:** Enhancing drug solubility using magnetic nanoparticles.

Drug/Compound	Nanocarrier Type	Size	Route	Effect or Remarks on Solubility/Bioavailability	Reference
Rifampicin and Thymopentin	Glyceryl Monostearate/Soybean Phosphatidylcholine Nanoparticles	150–200 nm	Inhalable Drug Delivery	Enhanced solubility and bioavailability in pulmonary systems.	[25]
Curcumin	PLA-HA/Fe_3_O_4_ Magnetic Nanoparticles	208 nm	Oral	Enhanced solubility and bioavailability in the GI tract, used for colorectal cancer treatment.	[26]
	Alginate/Chitosan-Functionalized Mn_0.8_Zn_0.2_Fe_2_O_4_ Nanoparticles	20 nm	Oral	Enhanced solubility, controlled release, and improved therapeutic efficacy against breast cancer cells.	[28]
	Magnetic Ferrite Nanoparticles Coated with BSA/Glycine Polymers	50–70 nm	Oral	Improved solubility in the GI tract, stable and sustained release profile, suitable for maintaining therapeutic levels.	[30]
Hydrocortisone	CS-g-PNVCL-Coated Fe_3_O_4_@SiO_2_ Core–Shell Nanoparticles	45–65 nm	Oral (GI-specific)	Improved solubility and therapeutic efficacy, with pH and temperature-sensitive release profile.	[27]
Celecoxib	Superparamagnetic Iron Oxide Nanoparticles (SPIONs)	15–25 nm	Oral	In situ amorphization under magnetic hyperthermia, increasing solubility by fivefold.	[29]
Docetaxel	Magnetic Nanostructured Lipid Carriers (MNLC)	120–150 nm	Oral	Increased solubility, reduced toxicity, and enhanced efficacy in lung cancer treatment.	[31]
N/A (this focuses on the delivery method rather than a specific drug)	Fe_3_O_4_ Nanoparticles Embedded in Liquid–Metal Soft Electronics	Diameter of robot legs: 1–2 mm	Oral	The robots are designed to traverse the GI tract effectively and deliver payloads in a minimally invasive manner through controlled magnetic navigation.	[33]
Methotrexate	Glucosamine-Modified Mesoporous Silica-Coated Magnetic Nanoparticles	100–150 nm	IV	Controlled release in tumor environment, efficient theranostic platform for cancer treatment.	[34]
Doxorubicin	Fe_3_O_4_-ßCD-Pep42-Coated Nanoparticles	17 nm	IV	Enhanced cancer cell uptake, reduced toxicity to healthy cells, combined use in imaging and therapy.	[35]
Silibinin	Magnetic Niosomal Nanoparticles (MNNPs)	50–70 nm	Oral	Controlled release, increased bioavailability, no significant toxicity to normal cells.	[36]
	Fe_3_O_4_ Nanoparticles (MR-SUFs)	10–100 nm	Oral	Enhanced GI retention and bioavailability.	[41]
5-Fluorouracil	Folic-Acid-Conjugated PEG-PCL-PEG-Coated SPIONs	100–150 nm	Oral	Targeted delivery in colon cancer, enhanced cellular uptake, controlled drug release at tumor sites.	[37]
Methotrexate	Glucosamine-Modified Mesoporous Silica-Coated Fe_3_O_4_	80–100 nm	IV	Enhanced targeting and controlled release of methotrexate.	[39]
Camptothecin	Ytterbium Ferrite/PLGA Superparamagnetic Hybrid	120–150 nm	IV	Enhanced delivery and reduced toxicity, used for targeted cancer therapy.	[43]

**Table 2:** Magnetic nanoparticles in disease treatment.

Disease Type	Type of MNPs	Models	Therapeutic Outcomes	Reference
Cancer (ferroptosis-based therapy)	Iron oxide nanoparticles	In vitro (cancer cells)	Enhanced ferroptosis in cancer cells, potential for image-guided therapy combining diagnostic imaging and ferroptosis to treat cancer.	[54]
Cancer (photothermal therapy)	Tannin-stabilized superparamagnetic iron oxide	In vitro (cancer cells)	Photothermal effect effectively generated hyperthermia, causing significant cancer cell death under laser light exposure.	[55]
Cancer (chemotherapy and hyperthermia)	Nylon-6 coated Fe_3_O_4_ nanoparticles with Doxorubicin	In vitro (A549, HEK 293FT cells)	pH-sensitive drug release and significant reduction in cancer cell growth, demonstrating potential for targeted delivery.	[56]
Cancer (breast cancer)	ECO/siRNA nanoparticles for DANCR lncRNA inhibition	In vivo (mouse model)	Significant inhibition of tumor growth with image-guided delivery, especially in highly aggressive tumors.	[57]
Melanoma	Paclitaxel-loaded lipid-coated Manganese ferrite	In vitro (B16F10 melanoma cells)	Dual chemo-magnetic hyperthermia therapy improved drug delivery and reduced systemic toxicity, with significant melanoma cell death.	[58]
Pancreatic cancer	Fe_3_O_4_ nanoparticles for hyperthermia	In vitro (pancreatic BxPC3 cells)	Combined hyperthermia and proton therapy reduced cancer cell survival and increased DNA damage, demonstrating synergistic therapeutic effects.	[59]
Neurological disorders	SPIONs	In vivo (mouse model)	SPIONs showed promise in treating Alzheimer’s disease by reducing amyloid β-protein clumping and protecting neurons from inflammation and oxidative stress. They also improved cognitive function and immunity by influencing the gut–brain connection.	[60]
Central nervous system Disorders	Magnetic hybrid nanovesicles	In vivo (various CNS models)	Enhanced diagnosis and treatment of CNS disorders by crossing the blood–brain barrier (BBB), with potential for improved drug delivery and reduced side effects.	[61]
Neurological disorders (Parkinson’s)	Magnetothermal nanoparticles	In vivo (mouse model)	Magnetothermal neuromodulation improved motor behavior in Parkinson’s disease models, offering a non-invasive alternative to deep brain stimulation.	[62]
Neurological disorders (Alzheimer’s)	SPIONs functionalized with transferrin	In vitro (brain cells)	Efficient isolation and targeting of brain-derived exosomes for diagnostic and therapeutic applications in neurodegenerative diseases.	[63]
Gastrointestinal disorders	SPIONs and quantum dots	In vivo (mouse model)	SPIONs were utilized to improve the diagnosis of colorectal cancer and inflammatory bowel diseases. Quantum dots enhanced imaging in CRC-bearing mice, allowing for better diagnosis and treatment planning.	[64]
Gastrointestinal cancers	Fe_3_O_4_ encapsulated with orange pectin	In vitro (HT-29, HCT 116, etc.)	Significant cytotoxicity against colorectal, pancreatic, and gastric cancer cells, with reduced side effects on healthy cells.	[65]
Cardiovascular diseases (CVDs)	Iron oxide nanoparticles (IONPs) functionalized with targeting ligands	In vitro	Enhanced imaging and therapy of cardiovascular conditions, including drug delivery and real-time monitoring of therapeutic responses, personalized treatment applications.	[68]
	SPIONs and CLIO nanoparticles	In vivo (various models)	SPIONs, combined with tissue plasminogen activator (tPA), significantly reduced clotting and improved blood flow in cardiovascular diseases. CLIO nanoparticles were developed for fibrin-targeting to improve clot dissolution in thrombotic diseases.	[70]
	Drug-loaded MNPs on flexible polyimide catheter	In vivo (rat model)	Effective targeted drug delivery to treat CVDs, demonstrated improved precision of drug release at the target site, reducing stent-induced complications like restenosis.	[71]

## Data Availability

Details are available from the authors.
